# Genome Organization of *Escherichia Phage* YD-2008.s: A New Entry to *Siphoviridae* Family

**DOI:** 10.21315/tlsr2018.29.1.3

**Published:** 2018-03-02

**Authors:** Dharmela Sellvam, Nyok Sean Lau, Yahya Mat Arip

**Affiliations:** 1 School of Biological Sciences, Universiti Sains Malaysia, 11800 USM Pulau Pinang, Malaysia; 2 Centre for Chemical Biology, Universiti Sains Malaysia, Sains@USM, Block B, No. 10, Persiaran Bukit Jambul, 11900 Bayan Lepas, Pulau Pinang, Malaysia

**Keywords:** *Siphoviridae*, Complete Genome Sequencing, *Hk578virus*, ICTV

## Abstract

Malaysia is one of the countries that are loaded with mega biodiversity which includes microbial communities. Phages constitute the major component in the microbial communities and yet the numbers of discovered phages are just a minute fraction of its population in the biosphere. Taking into account of a huge numbers of waiting to be discovered phages, a new bacteriophage designated as *Escherichia phage* YD-2008.s was successfully isolated using *Escherichia coli* ATCC 11303 as the host. *Phage* YD-2008.s poses icosahedral head measured at 57nm in diameter with a long non-contractile flexible tail measured at 107nm; proving the phage as one of the members of *Siphoviridae* family under the order of *Caudovirales*. Genomic sequence analyses revealed *phage* YD-2008.s genome as linear dsDNA of 44,613 base pairs with 54.6% G+C content. Sixty-two open reading frames (ORFs) were identified on *phage* YD-2008.s full genome, using bioinformatics annotation software; Rapid Annotation using Subsystem Technology (RAST). Among the ORFs, twenty-eight of them code for functional proteins. Thirty two are classified as hypothetical proteins and there are two unidentified proteins. Even though majority of the coded putative proteins have high amino acids similarities to phages from the genus *Hk578likevirus* of the *Siphoviridae* family, yet *phage* YD-2008.s stands with its’ own distinctiveness. Therefore, this is another new finding to *Siphoviridae* family as well as to the growing list of viruses in International Committee on Taxonomy of Viruses (ICTV) database.

## Abstract (ru)

Malaysia merupakan salah sebuah negara yang kaya dengan kepelbagaian biologi, termasuk komuniti mikrob. Faj adalah komponen utama di dalam komuniti mikrob tetapi bilangan faj yang ditemui sehingga kini hanyalah sebahagian kecil dari keseluruhan populasi faj dalam biosfera. Memandangkan jumlah faj yang belum diemui adalah banyak, satu faj baharu telah berjaya dipencilkan dengan menggunakan *Eschrichia coli* ATCC 11303 sebagai perumah. Faj baru ini dinamakan *Eschrichia phage* YD-2008.s. Faj ini mempunyai kepala berbentuk ikosahedra berukuran 57nm diameter dengan struktur ekor yang fleksibel dengan panjang berukuran 107nm diameter; membuktikan ia adalah dari *Siphoviridae family* dibawah *Caudovirales order*. Analisis jujukan genomik menunjukkan *phage* YD- 2008.s mempunyai genom dsDNA yang linear bersaiz 44,613bp dengan kandungan 54.6% G+C. Dengan menggunakan perisian anotasi bioinformatik (RAST), sebanyak enam puluh dua kerangka bacaan (ORFs) telah dikenal pasti dalam genom *phage* YD-2008.s. Diantara ORFs yang dikenalpasti, dua puluh lapan dikodkan mempunyai protein berfungsi. Malah, 32 ORFs diklasifikasi sebagai protein hipotetikal dan dua lagi ORF baru belum dikenal pasti. Walaupun, majoriti protein berkod yang diduga menunjukkan persamaan asid amino yang tinggi dengan faj dari genus ‘HK578likevirus’ dari *Siphoviridae family*, tetapi *phage* YD-2008.s mempunyai keunikan tersendiri. Oleh itu, faj ini merupakan satu lagi penemuan baharu dalam *Siphoviridae family* serta tambahan virus baru di dalam pangkalan data Jawatankuasa Antarabangsa Taksonomi Virus (ICTV).

## INTRODUCTION

*Escherichia coli* is one of the well studied gram negative bacteria. *E. coli* strains are group of bacteria that are commonly found which are important to microbiology, as well as, biotechnology field (Abeles & Pride 2014). Among of its importance in microbiology would include serving as the hosts for many phages. In fact, the well studied groups of phages are those infecting *E. coli* (Dessel *et al.* 2005). Phages are non-pathogenic viruses to human, animals and plants. Phages are very specific to bacteria or in other words bacteria are the natural preys to bacteriophages (Duck & Park 2012; Klumpp & Loessner 2013). Due to its host specificity, phages are excellent agents to control bacteria populations, as well as, maintaining bacteria diversities (Chang & Kim 2011). In phage therapy, phages show very promising results in dealing with multi-resistant antibiotic bacteria (Chang & Kim 2011; Cannon *et al.* 2013; Klumpp & Loessner 2013). Being the most abundant biological entity on biosphere with an estimation of 10^31^ particles (Ackermann 1998; Li *et al.* 2010) and nanometre measured sized microbe phages have been used as useful model systems in various molecular research work. Besides, phages are ubiquitous and could be found wherever hosts reside. Thus, this would make them as diversed as their hosts (Ackermann 1998; Valera *et al.* 2014; Yu *et al.* 2016). Even though phages are huge in number but the total number of phages that have been discovered and examined under electron microscope are only in the range of 6,300. Over 96% of discovered phages are tail phages, under the order *Caudovirale* and thus formed the biggest group in prokaryote viruses (Ackermann 1998; Ackermann & Prangishvili 2012; Yu *et al.* 2016). Tail phages are divided into three major families: *Siphoviridae* (57.3%), *Myvoviridae* (24.8%) and *Podoviridae* (14.2%) as reported in Ackermann and Prangishvili (2012).

Isolation and propagation of novel phages are facilitated by phage abundances in the biosphere. However, to fully characterise the phage isolate up until the genomic level is costly and time consuming (Valera *et al.* 2014). Nevertheless, to advance our understanding on phages and their bacteria hosts’ evolution, then, the necessity for phage genome sequencing is vital. Therefore, the development of a high throughput, next generation sequencing (NGS) technology in 2005 has given the chances to virologists to identify more of those yet to be revealed phages (Forrester & Hall 2014; Valera *et al.* 2014). As of August 2015, more than 1500 phage genomes have been completely sequenced and deposited in the National Centre for Biotechnology Information (NCBI) database, compared to the year 2007 where only ~400 phage genomes were deposited in NCBI GenBank (Savalia *et al.* 2008). Therefore, without doubt, there are a lot more phages are waiting to be discovered and characterised. This paper would discuss on a new phage that has been designated as *Escherichia phage* YD-2008.s. The phage was successfully isolated from goat faeces using *E.coli* ATCC 11303 as the host and its genome was fully sequenced with the aid of MiSeq system (Illumina). *Phage* YD-2008.s full genome sequence could be accessed from NCBI GenBank using accession No. KM896878.1 or accession No. NC_027383.1.

## MATERIALS AND METHODS

### Isolation and Propagation of Phage

Fresh goat faecal samples were collected from a goat farm in Kampung Batu Putih, Balik Pulau, Penang, Malaysia (GPS coordinates: latitude – 5° 24′ 18.515″; longitude – 100° 12′ 17.254″). Phage isolation was carried out according to previous report but with modifications (Chang *et al.* 2011). Briefly, 20 g of goat faeces was mixed with 100 ml of TS buffer (8.5 g NaCl and 1 g tryptone per liter). A volume of 10 ml overnight *E*. *coli* ATCC 11303 culture was mixed with 10 ml of faeces suspension and added into 20 ml of double strength LB broth (20 g bactotryptone, 10 g yeast extract and 10 g NaCl per liter). The mixture was incubated at 37°C with shaking at 160 rpm for 24 hours to enrich phage population. Subsequently, large debris was filtered through a filter paper and the filtrate was centrifuged at 4000 × *g* for 15 minutes. The filtrate was passed through a 0.45 μm syringe filter (Minisart, Sartorius Stedim Biotech, Germany) and stored at 4°C. The filtrate was used in standard protocol of soft agar overlay method (Pearson 2013) with *E*. *coli* ATCC 11303 as the host for phage isolation. Plaques (clear zone) that formed on the soft agar overlay were due to the lysis of the bacterial cells infected by the phages. Following that, phages were purified using single plaque purification method as describe previously (Jones & Portnoy 1994).

### Transmission Electron Microscope (TEM)

Phage observation using transmission electron microscope (TEM) (Philips CM12 equipped with analysis system, Philips Electron Optics) was according to Ackermann (2007) with modifications. A drop of phage sample (approximately 5 × 10^10^ pfu/ml) was applied onto carbon-coated grid (400 mesh copper grid) and left for three to five minutes. Then, a drop of 2% methylamine tungstate was applied to negatively stain the phage sample and after one minute. The prepared phage sample was ready to be viewed under TEM (Philips CM12 equipped with analysis system, Philips Electron Optics).

### Phage Genome Extraction

The phage genome was extracted using phenol: chloroform technique as reported previously (Sellvam & Arip 2012). A volume of 2 μl of RNase A and DNase I each at final concentration of 1 mg/mL were added into phage sample and incubated at 37°C for 30 min. After incubation phage genome was extracted with phenol:chloroform:isoamyl solution (25:24:1). Then, the phage genome was precipitated by adding ice cold isopropanyl alcohol and incubated at −20°C overnight. After an overnight incubation, the mixture was spin at 14,000 rpm for 20 minutes at room temperature. The supernatant was decanted and the pellet was washed with 0.7 mL of ice cold 70% ethyl alcohol. Following centrifugation at 14,000 rpm for 10 min, the pellet was air-dried and dissolved in 30 μl of nuclease free water and stored at −20°C.

### Complete Genome Sequencing

The phage genome was sequenced using Next Generation Sequencing (NGS) method using MiSeq Illumina technology. Briefly, in the process of sequencing, Nextera XT DNA sample preparation kit (Illumina) was used to prepare the purified genome sample and sequenced was proceed using paired end 2 X 150 bp reads on the MiSeq system (MiSeq reagent; Nano kit v2, 300 cycles). The data was analysed using the Assembly workflow of the MiSeq reporter for quality control purpose.

### Bioinformatics Analysis

The paired-end reads obtained by Illumina MiSeq sequencing were assembled into a full genome sequence using *de novo* assembly of CLC Genomic Workbench 6.0 (CLC Bio, Denmark) and compared using Geneious R8 as well. The length of phage YD-2008.s genome, formation of DNA, GC contents and nucleotide (A, T, C, G) compositions details were obtained from CLC Genomic Workbench 6.0. Predictions of open reading frames (ORFs) were carried out using GeneMark. hmm v3.25 (Chang *et al.* 2011; Lee *et al.* 2013) and confirmed with RAST server (Chang & Kim 2011). Nucleotide and protein sequences were subjected to search against NCBI BLAST (Basic Local Alignment Search Tool) tools to search for homologues similarities as described in previous work (Pan *et al.* 2013; Yu *et al.* 2016). The complete genome sequence of *phage* YD-2008.s was subjected to BLASTn against non-redundant nucleotide collection (nr/nt) (https://blast.ncbi.nlm.nih.gov/Blast; accessed in 15 August 2014). Each of the predicted ORFs functions’ were cross checked against non-redundant protein databases; BLASTX, BLASTP, as well as, BLAST against phage proteins database on with cut off e-value > 10^−5.^ (https://blast.ncbi.nlm.nih.gov/Blast.cgi?PROGRAM=blastx; https://blast.ncbi.nlm. nih.gov/Blast.cgi?PROGRAM=blastp, accessed in September 2014) (Dessel *et al.* 2005; Chang & Kim 2011; Lee *et al.* 2013; Pearson 2013).

### Nucleotide Sequence Accession Number

The complete genome sequence of *Escherichia phage* YD-2008.s was deposited in GenBank under (Accession No. KM896878.1 or NC_027383.1).

## RESULTS AND DISCUSSION

### Isolation and Morphological Study of *Escherichia phage* YD-2008.s

**Figure 1:**
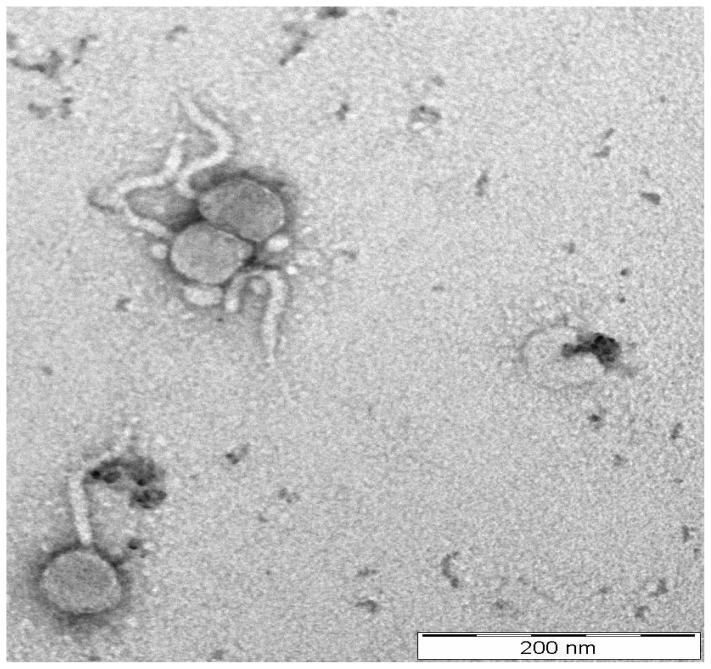
Transmission electron micrographs (TEM) of Escherichia phage YD-2008.s. The purified phage sample was negatively stained with 2% of methyl tungstate [3]. The phage has an icosahedral head of 57nm diameter and a noncontractile long tail of approximately 107nm. The scale bar represents 200nm.

*Phage* YD-2008.s was isolated from goat faeces in Penang, Malaysia, using *Escherichia coli* ATCC 11303 as the host. The phage poses typical features belonging to *Siphoviridae* family of *Caudovirales* order. Phages in this family would have a capsid with diameter about 50–60 nm and long non-contractile tail that could reach up to 750 nm in length (Matsuzaki *et al.* 2005; King *et al.* 2012). The transmission electron micrograph (TEM) pictures show that *phage* YD-2008.s has an icosahedral capsid measured 57nm in diameter with a flexible long non-contractile tail measured at 107nm in length (Fig. 1).

### Basic Genomic Identity of *Escherichia phage* YD-2008.s

The complete genome of *phage* YD-2008.s was successfully sequenced using NGS method. Paired reads assembly with *de novo* assembly tool (CLC Bio 6.0, Denmark) indicates that *phage* YD-2008.s is a linear double stranded DNA. The phage composes of 44,613 base pairs with nucleotide composition of A (22.2%), T (23.2%), G (26.8%) and C (27.8%). Overall, it has G+C content of 54.6%, which is a slightly higher than G+C content found in other *E.coli* phages (50–51%) (Matsuzaki *et al.* 2005; Li *et al.* 2010; Chang & Kim 2011). However, the G+C content of *phage* YD-2008.s is very much similar to phages from genus *Hk578virus*, a member of *Siphoviridae* family.

**Figure 2:**
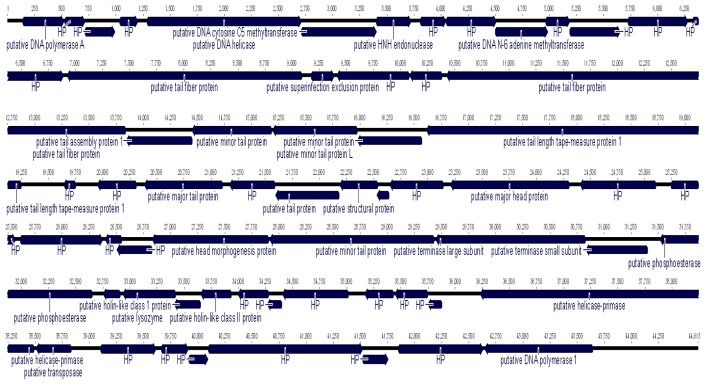
Schematic representation of the linear dsDNA genome of *Escherichia phage* YD- 2008.s; 62 predicted proteins are represented by arrows.

Bioinformatics studies on full genome of *phage* YD-2008.s have identified a total number of 62 ORFs, where 96.8% of them encoded for functional and hypothetical proteins. The range of the ORFs is between 39–1139 amino acids (aa) with an average of 216 aa. Majority of the ORFs are located on the bottom strand with 62.9% of them and the balance of 37.1% of the ORFs is coded on the upper strand. Each of the coding DNA sequence (CDS) function was subjected to BLASTP programme and re-confirmed with NCBI BLAST against phage protein programme as well. Among the sixty-two ORFs, 28 putative coded proteins were annotated with known functions. Thirty-two were conserved hypothetical proteins which shared similarities to unknown function of other registered phage proteins in NCBI database. Genome of *phage* YD-2008.s also contain two ORFs with no hits (no significant similarity search) against the NCBI non-redundant protein database (BLASTP) and BLAST phage protein database. The two ORFs could be coded for new phage proteins or might derived from the host genome. The complete details of each of the predicted coding ORFs of *phage* YD-2008.s are listed in Table 1 with the schematic picture shown in (Fig. 2). Majority of the ORFs show confident hits against proteins of the phages from genus *Hk578virus* with 76–100% amino acids identity at E-value cut off >10^−5^. Hence, *Escherichia phage* YD-2008.s could be grouped together with the member of genus *Hk578virus*, another new phage in *Siphoviridae* family (Adriaenssens *et al.* 2014).

### Sequence Analysis of Predicted Proteins of *Escherichia phage* YD-2008.s

A total of 45% of the CDS from the full genome of *phage* YD-2008.s hit sequence similarities to known proteins with known molecular functions. These predicted proteins of *phage* YD-2008.s could be categorised into five main functional groups; structural, DNA replication and recombination, lysis, packaging and additional functions including host interaction and nucleotide metabolism. For the structural proteins, *phage YD*-2008.s composes of all essential proteins typically found including tail fiber protein, tail assembly protein, minor tail protein, tail length tape-measure protein 1, major tail protein, structural protein and major head protein. Most of the proteins coded for structure components are arranged at middle of the genome and all of them are on the bottom strand in reverse orientation. The major capsid protein (ORF33) and the major tail protein (ORF27) have 366 and 214 of amino acid respectively. Whereby, the tail fiber protein (ORF19) has the largest size gene with 1139 amino acids among all the coded ORFs.

The phage encodes proteins, such as DNA polymerase, helicase, endonuclease and helicase-primase for DNA replication/recombination process. It also encodes few proteins categorised under nucleotide metabolism that includes DNA cytosine C5 methyltransferase, DNA N-6 adenine methyltransferase, phosphoesterase and transposase. Proteins that encodes for DNA replication/recombination and nucleotide metabolism that are resided in phage genome are produced with the aid of host’s machinery since phages are known to be host dependent living microorganisms (Savalia *et al.* 2008; Li *et al.* 2010; Chang & Kim 2011).

Theoretically, phages need to lyse their host cells in order to release their progeny virions out of the bacteria intracellular as the final step of the virus life cycle. For this purpose they need to have encoded lysis protein (Li *et al.* 2010; Gan *et al.* 2013; Klumpp & Loessner 2013). To accomplish this function *phage YD*-2008.s has proteins that encode for lysozyme, holin-like class I protein and holin-like class II protein. These lysis encoded genes lies next to each other in the genome. Furthermore, these proteins substantiate that *phage YD*-2008.s is a lytic phage. The phage genome also encodes proteins for packaging as well, such as head morphogenesis protein, terminase large subunit and terminase small subunit where all of these proteins are needed for the assembly of viral particles.

Besides, *phage* YD-2008.s poses host interaction protein denoted as superinfection exclusion protein. This protein would prevent secondary infections from other phages of the closely related family. This viral protein has the capability to prevent the entry of DNA from other phages or modify the entry receptor on the host cell. Hence, the infected host would be dominated with only a specific phage at a time (Chumby *et al.* 2012; Lee *et al.* 2013). The predicted ORFs that coded for functional phage proteins are summarised into different protein clusters as tabulated in Table 2.

## CONCLUSION

To conclude, we have sequenced and analysed a lytic phage; *Escherichia phage* YD-2008.s of *E.coli* ATCC 11303 that was isolated from goat faeces. Although nucleotide search against (BLASTn) of the whole genome of *phage YD*-2008.s shared 91–94% DNA similarity with phages from genus *Hk578viruses* but there are evidences to prove that *phage YD*-2008.s has its own identity made it different from phages of the same genus. The *Escherichia phage* YD-2008.s morphological dimension, complete genome length, number of ORFs, the genes arrangement in the genome and host are different from *Hk578likevirus*. In addition, *phage YD*- 2008.s does not share 100% DNA similarity with any other registered phages in NCBI database as well as, International Committee on Taxonomy of Viruses (ICTV) database. So far, there are five phages under the genus of *Hk578virus*: *Enterobacteria phage* SSL-2009a (Accession No. FJ750948.2) shared 94% DNA similarity with *phage* YD-2008.s, *Enterobacteria phage* JL1 (Accession No. JX865427.2) shared 94% DNA similarity with *phage* YD-2008.s, *Enterobacteria phage* Hk578 (Accession No. JQ86375.1) shared 92% DNA similarity with *phage* YD-2008.s, *Shigella phage* Ep23 (Accession No. JN984867.1) shared 92% DNA similarity with *phage* YD-2008.s and *Sodalis phage* SO-1 (Accession No. GQ502199.1) shared 91% DNA similarity with *phage* YD-2008.s (King *et al.* 2012; McNair *et al.* 2012; Adriaenssens *et al.* 2014). Therefore, *Escherichia phage* YD- 2008.s (Accession No. KM896878.1) could be claimed as new phage in genus of *Hk578viruses* of *Siphoviridae* family. It will be another new finding to *Siphoviridae* family that contribute to the growing list of phages in ICTV database since there are still a lot of phages waiting to be discovered.

## Figures and Tables

**Table 1:** ORFs of *Escherichia phage* YD-2008.s and predicted protein functions.

Coding ORFs	Strand	Start	Stop	Length aa	**Predicted functions	E-value/% aa identity
1	+	140	523	127	putative DNA polymerase A [gp38 Sodalis phage SO-1]	4e-81/100
2	+	528	719	63	hypothetical protein [Enterobacteria phage SSL-2009a]	2e-32/94
3	+	716	994	92	hypothetical protein [Enterobacteria phage HK578]	2e-58/100
4	+	1035	1208	57	hypothetical protein [gp32 Sodalis phage SO-1]	1e-20/100
5	+	1289	2713	460	putative helicase [Enterobacteria phage SSL-2009a]	0.0/99
6	+	2710	3405	231	putative DNA cytosine C5 methyltransferase [gp32 Sodalis phage SO-1]	4e-157/98
7	+	3402	3725	107	putative HNH endonuclease[gp31 Sodalis phage SO-1]	2e-71/98
8	+	3807	4049	80	hypothetical protein [Enterobacteria phage SSL-2009a]	7e-48/93
9	+	4050	4514	154	hypothetical protein [Enterobacteria phage SSL-2009a]	5e-72/84
10	+	4501	4986	161	putative DNA N-6 adenine methyltransferase [gp29 Sodalis phage SO-1]	6e-115/99
11	+	4967	5191	74	hypothetical protein [Shigella phage EP23]	1e-40/91
12	+	5188	5658	156	hypothetical protein [Enterobacteria phage JL1]	2e-112/100
13	+	5723	6277	184	hypothetical protein [Enterobacteria phage HK578]	7e-123/95
14	+	6320	6895	191	hypothetical protein [Enterobacteria phage HK578]	1e-135/98
15	−	9093	6922	723	putative tail fiber protein [Escherichia phage bV_EcoS_AKFV33]	2E-133/57
16	+	9174	9392	72	putative superinfection exclusion protein [Enterobacteria phage SSL-2009a]	5e-44/94
17	−	10081	9410	223	hypothetical protein [Enterobacteria phage SSL-2009a]	4e-155/98
18	−	10386	10084	100	hypothetical protein [Enterobacteria phage SSL-2009a]	6e-62/98
19	−	13843	10424	1139	putative tail fiber protein [Enterobacteria phage SSL-2009a]	0.0/99
20	−	14457	13840	205	putative tail assembly protein 1 [Enterobacteria phage JL1]	9e-123/99
21	−	15188	14448	246	putative minor tail protein [Enterobacteria phage SSL-2009a]	9e-180/98
22	−	15979	15191	262	putative minor tail protein L [Enterobacteria phage JL1]	0.0/99
23	−	16575	15976	199	putative minor tail protein [Enterobacteria phage T1]	7e-11/61
24	−	19252	16610	880	putative tail length tape-measure protein 1 [Enterobacteria phage JL1]	0.0/99
25	−	19753	19637	38	hypothetical protein [Enterobacteria phage HK578]	3e-18/100
26	−	20313	19951	120	hypothetical protein [Enterobacteria phage JL1]	7e-83/100
27	−	21108	20383	214	putative major tail protein [Enterobacteria phage JL1]	2e-154/97
28	−	21592	21170	140	hypothetical protein [Shigella phage EP23]	4e-94/97
29	−	22185	21592	197	putative tail protein [Shigella phage EP23]	2e-138/98
30	−	22540	22187	117	putative structural protein [Enterobacteria phage JL1]	1e-75/97
31	−	22646	22527	39	*unknown gene	-
32	−	23148	22648	166	hypothetical protein [Enterobacteria phage JL1]	4e-106/92
33	−	24307	23207	366	putative major head protein [Enterobacteria phage HK578]	99
34	−	25106	24405	233	hypothetical protein [Enterobacteria phage SSL-2009a]	6e-140/99
35	+	25246	25575	109	hypothetical protein [Enterobacteria phage SSL-2009a]	6e-58/97
36	+	25615	26379	254	hypothetical protein [Enterobacteria phage SSL-2009a]	0.0/96
37	−	26552	26394	52	hypothetical protein [Shigella phage EP23]	1e-28/100
38	−	26835	26506	109	hypothetical protein [Enterobacteria phage JL1]	1e-54/97
39	−	27907	26828	359	putative head morphogenesis protein [Enterobacteria phage SSL-2009a]	0.0/97
40	−	29435	27915	506	putative minor tail protein [gp3 Sodalis phage SO-1]	0.0/98
41	−	30832	29447	461	putative terminase large subunit [gp2 Sodalis phage SO-1]	0.0/98
42	−	31404	30832	190	putative terminase small subunit [ gp1 Sodalis phage SO-1]	1e-94/99
43	−	32656	31514	380	putative phosphoesterase [Shigella phage EP23]	0.0/98
44	−	32914	32753	53	*unknown gene	-
45	−	33422	32931	163	putative lysozyme [Shigella phage EP23]	3e-114/100
46	−	33654	33409	81	putative holin-like class I protein [Enterobacteria phage JL1]	3e-48/98
47	−	33941	33651	96	putative holin-like class II protein [Shigella phage EP23]	3e-60/99
48	−	34281	33994	95	hypothetical protein [Enterobacteria phage HK578]	2e-46/97
49	−	34406	34266	46	hypothetical protein [Enterobacteria phage SSL-2009a]	4e-25/98
50	−	35020	34403	205	hypothetical protein [Enterobacteria phage SSL-2009a]	2e-129/88
51	−	35435	35166	89	hypothetical protein [Enterobacteria phage JL1]	6e-54/93
52	−	35750	35445	101	hypothetical protein [Enterobacteria phage HK578]	3e-56/89
53	−	35881	35747	44	hypothetical protein [Enterobacteria phage SSL-2009a]	9e-11/76
54	−	38494	36230	754	putative helicase-primase [Enterobacteria phage JL1]	0.0/99
55	−	38834	38505	109	putative transposase [gp47 Sodalis phage SO-1]	4e-68/95
56	+	39104	39616	170	hypothetical protein [Enterobacteria phage HK578]	1e-16/100
57	+	39665	39916	83	hypothetical protein [Enterobacteria phage SSL-2009a]	4e-52/99
58	+	39916	40095	59	hypothetical protein [Enterobacteria phage HK578]	8E-34/97
59	+	40095	41525	476	hypothetical protein [Enterobacteria phage JL1]	0.0/99
60	+	41518	41760	80	hypothetical protein [Enterobacteria phage SSL-2009a]	4e-47/91
61	+	41851	42633	260	hypothetical protein [Enterobacteria phage HK578]	2e-108/97
62	−	43647	42646	333	putative DNA polymerase 1 [Enterobacteria phage SSL-2009a]	0.0/99

* no significant similarity search

** Predicted functions of the deduced hypothetical/putative proteins were made against the following databases: RAST, NCBI and GeneMark.

**Table 2:** Functional groups of putative genes in *Escherichia phage* YD-2008.s

Protein cluster	ORF number
Structural gp	15, 19, 20, 21, 22, 23, 24, 27, 29, 30, 33, 40
DNA replication/recombination gp	1, 5, 7, 54, 62
Nucleotide metabolism gp	6, 10, 43, 55
Lysis gp	45, 46, 47
Packaging gp	39, 41, 42
Host interaction gp	16
